# When meta-analysis misleads: the need for methodological integrity in e-cigarette research

**DOI:** 10.1007/s11739-025-04019-w

**Published:** 2025-06-17

**Authors:** Riccardo Polosa, Giulio Geraci, Yusuff Adebayo Adebisi

**Affiliations:** 1 Center of Excellence for the Acceleration of HArm Reduction (CoEHAR), University of Catania, Via S. Sofia, 78-Ed. 4, P. 2, 95123 Catania, Italy; 2 Department of Clinical & Experimental Medicine, University of Catania, Catania, Italy; 3 Faculty of Medicine and Surgery, “Kore” University of Enna, 94100 Enna, Italy; 4 College of Social Sciences, University of Glasgow, Glasgow, UK

## Introduction

Tobacco harm reduction (THR), especially in the context of electronic cigarette (EC) research, remains highly contested within public health, often due to studies compromised by methodological flaws [1–3]. These shortcomings in study design, analysis, terminology, or interpretation can distort scientific record, erode public trust, and generate misleading or uncritical media coverage and policy reports [4–6]. These challenges become especially consequential when flawed evidence is aggregated and amplified through meta-analyses, which play a central role in shaping policy and clinical guidance.

In this issue of *Internal and Emergency Medicine*, Rodu and colleagues [7] critically assess a widely cited 2024 meta-analysis by Glantz et al. [8], published in *NEJM Evidence*. Glantz and coauthors concluded that e-cigarette use is associated with disease odds similar to those of cigarette smoking for cardiovascular conditions, and still substantial, though lower, for asthma, COPD, and oral diseases. Rodu et al. identify major methodological flaws that call these conclusions into question. This underlines the urgent need for rigorous and transparent evidence synthesis in tobacco harm reduction science.

## Dangers of garbage in, garbage out

The credibility of any meta-analysis is directly dependent on the quality, comparability, and methodological rigor of the studies it includes [9]. When foundational studies are flawed or inconsistent, the meta-analysis built upon them is likely to be compromised, regardless of statistical sophistication [9]. This principle, often summarized by the phrase “garbage in, garbage out”, is particularly relevant in the case examined by Rodu and colleagues. Their critique of the meta-analysis by Glantz et al. exposes a series of methodological issues that raise serious concerns about the reliability of the conclusions.

One of the most problematic aspects highlighted by Rodu et al. is the indiscriminate aggregation of disease outcomes into excessively broad diagnostic categories. For example, Glantz et al. [8] grouped vastly different conditions, such as erectile dysfunction and myocardial infarction, under the umbrella of “cardiovascular disease.” Similarly, Glantz et al. grouped influenza (classified as “respiratory symptoms”) and chronic obstructive pulmonary disease (COPD) within a single category for pooled analysis, despite having distinct clinical profiles. As a result, the pooled risk estimates become difficult to interpret and may give a distorted view of the potential health risks associated with e-cigarette use. This lack of clinical coherence introduces systematic bias and reduces the validity of any generalized conclusions drawn from the analysis.

Adding to this concern is the heavy reliance on cross-sectional studies, which accounted for the majority, 76 percent (94 out of 124), of the odds ratios included in the Glantz et al. meta-analysis. Cross-sectional designs assess both exposure and outcome at a single point in time [10], which means they are inherently incapable of establishing whether vaping preceded the onset of disease. Without a clear temporal sequence, the evidence cannot support causal inferences.

Many of the studies included in the Glantz et al. meta-analysis relied on data from large-scale surveys, such as the National Health Interview Survey (NHIS) and the Behavioral Risk Factor Surveillance System (BRFSS). While these datasets are valuable for descriptive epidemiology, they typically lack crucial temporal information, such as the age of smoking or vaping initiation and the timing of disease diagnosis. Without this temporal resolution, it becomes impossible to establish whether the exposure could plausibly have contributed to the health outcome. We have previously highlighted the gravity of this issue [11], stressing that the persistent repetition of such methodological shortcomings has now reached a scale that risks undermining the credibility of public health science itself. A further concern relates to the possibility of double-counting results derived from the same data sources (e.g., NHIS, BRFSS, or PATH), particularly for outcomes like COPD, which were reported in multiple studies using overlapping samples. Although Glantz et al. report inflating standard errors to account for potential correlation between estimates, this statistical correction does not eliminate the risk that individual disease cases were counted more than once across pooled estimates, which could artificially enhance the perceived consistency or precision of the findings.

A related limitation is the near-total absence of cumulative exposure metrics across the studies included in Glantz et al.’s meta-analysis. As highlighted in recent methodological guidance [3], observational studies should report dose–response exposure histories to avoid misclassification and residual confounding. When meta-analyses include studies lacking this level of granularity, they risk pooling data from participants with vastly different exposure intensities. This can obscure true dose–response relationships and conflate light, short-term use with heavy, chronic use. In the context of comparing exclusive and dual use, such exposure misclassification may seriously distort conclusions about relative harm.

## A closer look at the longitudinal evidence

Longitudinal studies, by virtue of their design, offer a stronger foundation for causal inference than cross-sectional analyses. These studies follow individuals over time, allowing researchers to observe how exposures such as e-cigarette use may precede or coincide with the development of health outcomes. In theory, this temporal sequencing provides a clearer understanding of potential cause-and-effect relationships. However, as Rodu and colleagues reveal in their detailed re-examination of the literature, even longitudinal studies can fall short of their potential when critical design elements are neglected.

While longitudinal studies are often considered superior to cross-sectional ones for establishing temporal sequence, it is important to avoid overgeneralization. A well-designed cross-sectional study, particularly one that captures the timing of both exposure and diagnosis, can still provide valuable insights. Conversely, prospective studies are not immune to flaws. Some, like the Goldberg-Scott et al. study heavily cited in the Glantz meta-analysis, suffer from methodological weaknesses, including inadequate exposure characterization and failure to control for key confounders. The strength of a study ultimately depends not on its design label, but on how carefully its methods are implemented and reported.

A central concern raised by Rodu et al. is the widespread failure of the longitudinal studies included in Glantz et al.’s meta-analysis to account for changes in smoking or vaping behavior during the follow-up period. This oversight is far from trivial. Nicotine use is dynamic, and many individuals transition between different forms of use over time. For instance, a participant may begin the study as someone who has quit smoking and currently vapes, but may later relapse into cigarette smoking only, or adopt dual-use habits. Without accounting for these behavioral shifts, any associations observed between exposure and disease outcomes may be confounded or entirely misattributed. Ignoring these transitions undermines the validity of the conclusions drawn from such data.

One notable exception in the literature is the study by Berlowitz et al. [12], which Rodu et al. highlight as a more methodologically sound investigation. This study rigorously adjusted for changes in smoking status and examined incident cardiovascular disease over time. Crucially, it found no statistically significant association between e-cigarette use and cardiovascular outcomes. This finding directly challenges the overarching conclusions of Glantz et al.’s meta-analysis and raises important questions about the validity of pooling disparate studies without carefully considering their methodological integrity.

The case of Xie et al.’s study [13] further illustrates the perils of residual confounding. Xie et al. reported a statistically significant association between e-cigarette use and the development of chronic obstructive pulmonary disease (COPD). However, upon reanalysis, Rodu and colleagues reveal a crucial detail: nearly all of the individuals diagnosed with COPD in the study were either current or former smokers. In fact, only one participant with COPD had never smoked. This insight suggests that the observed link between vaping and COPD likely reflects the long-term effects of prior tobacco smoking, rather than any independent effect of e-cigarette use. This form of confounding, where the influence of a key variable like smoking history is insufficiently accounted for, can seriously distort interpretations and lead to inappropriate attributions of harm.

More recent high-quality evidence lends additional support to these concerns. An umbrella review encompassing twelve systematic reviews concluded that there is no convincing evidence of short- or medium-term respiratory harm associated with e-cigarette use [14]. These systematic reviews were carefully selected based on their methodological rigor and comprehensiveness. Furthermore, when attention is restricted to studies involving individuals who have never smoked conventional cigarettes, the picture becomes even clearer. An expert narrative review [15], along with a separate systematic review [16], examined health outcomes specifically among never-smoking e-cigarette users. Both reviews found no evidence of serious or sustained respiratory harm attributable to vaping in this population. In parallel, a large international cohort study focused on exclusive e-cigarette users who had never smoked cigarettes [17] reported no clinically meaningful differences in respiratory symptoms compared to individuals who did not use any nicotine products. This study, which utilized validated respiratory symptom scales and followed participants over time, represents some of the most robust data currently available. It adds to a growing body of evidence that questions the validity of broad generalizations about vaping-related respiratory risk, particularly when these claims are based on studies that fail to properly isolate vaping as an independent exposure.

Nonetheless, it is important to acknowledge that many of the disease outcomes considered in meta-analyses such as Glantz et al.’s—particularly cardiovascular and chronic respiratory conditions—tend to occur in older populations, where prior smoking is common and findings from never-smoker cohorts may have limited generalizability.

Another major limitation in many of the included studies, including those cited by Glantz et al., is the treatment of dual use as a homogeneous category. Yet, someone who smokes daily and vapes occasionally may have a vastly different exposure profile, and risk, than someone who vapes daily but smokes only intermittently. Most analyses do not disaggregate these patterns, undermining meaningful interpretation of dual-use risk. More broadly, to our knowledge, no prospective study has followed a cohort of disease-free smokers over time while carefully tracking their transition to continued smoking, switching, dual use, or complete cessation—with adequate adjustment for baseline smoking history and confounding. Such a study design is essential for making valid inferences about the comparative risks of these trajectories and is currently underway [18, 19].

Together, these findings suggest that many of the alarming conclusions drawn were overstated. This is especially true when the analyses fail to disentangle the effects of vaping from those of smoking, a mistake that continues to plague much of the current literature. Going forward, it is imperative that longitudinal studies incorporate detailed, time-varying measures of exposure, and that analyses are appropriately adjusted to reflect the complex behavioral histories of nicotine users. This includes accounting for age at smoking initiation, duration and intensity of past tobacco use, and time since cessation—particularly because residual risks for diseases, such as COPD and cardiovascular disease, can persist for more than a decade after quitting. For example, relative risks for COPD may remain elevated for 10 years or more after cessation, especially in former heavy smokers [20]. Dual users may also differ systematically from exclusive users in baseline smoking history and level of nicotine dependence. Without such adjustments, studies risk misattributing legacy harms from smoking to e-cigarette use, leading to distorted conclusions about relative risk.

## The illusion of significance and the abuse of statistics

In addition to methodological concerns regarding study selection and exposure classification, Rodu and colleagues identify a range of statistical errors in Glantz et al.’s meta-analysis that further undermine its credibility. These issues are not limited to technical oversights; they reflect a problem of misapplied statistical reasoning presented as rigorous science despite key methodological concerns. When complex methods are used inappropriately or without transparency, they can lend an unwarranted sense of credibility to flawed conclusions.

One of the most concerning examples highlighted by Rodu and colleagues is the use of a Bonferroni correction to adjust for multiple comparisons. While statistical corrections are important tools to limit false-positive findings in studies with multiple tests, their application must be grounded in a clear theoretical or conceptual rationale. In this case, the use of the Bonferroni correction appeared arbitrary and lacked an adequate explanation of which comparisons it was meant to control for or why such a stringent adjustment was appropriate [21]. Without this clarity, the correction may distort the interpretation of results rather than enhance their reliability.

Additionally, the use of Bonferroni correction in this context is methodologically questionable. Bonferroni is designed to control the family-wise error rate by dividing the significance threshold (e.g., α = 0.05) by the number of comparisons. While this reduces the risk of Type I errors, it also reduces statistical power, especially in settings where multiple related outcomes are being tested. This makes Type II errors more likely, potentially masking true effects. Moreover, Bonferroni assumes that all tests are independent; an assumption that rarely holds in meta-analyses, where outcomes or subgroups often share correlated structures. As a result, applying Bonferroni in this setting is not only overly conservative but also conceptually inappropriate, doing little to resolve the deeper issue of overlapping datasets and potential case duplication.

Another major shortcoming was the failure of the meta-regression analysis to properly account for differences in study design. Meta-regression is a technique intended to explore the influence of study-level characteristics, such as sample size, methodology, or population, on the overall effect estimate [22]. Beyond study design, meta-regression offers a powerful tool to investigate why study results differ and to test hypotheses about potential effect modifiers or sources of bias. For example, Glantz et al. could have used meta-regression to examine whether associations varied by age group, duration of e-cigarette use, quality rating, or whether studies adjusted for cumulative smoking history. These analyses could have helped identify sources of heterogeneity and clarified whether certain subgroups were driving the observed effects. However, in the Glantz et al. analysis, this tool was not used to meaningfully differentiate between cross-sectional and longitudinal studies. This omission is critical because these study designs carry fundamentally different implications for interpretation. Lumping them together, or failing to explore how their findings differ, can mask important heterogeneity and create the illusion of coherence where none exists.

A particularly concerning statistical error identified by Rodu et al. was the misinterpretation of a non-significant p value as evidence that no difference exists between study designs. In scientific inference, a non-significant result indicates that the data do not provide strong enough evidence to reject the null hypothesis [23]. It does not, however, confirm that the null hypothesis is true. Mistaking a lack of statistical significance for evidence of equivalence is a common but serious error. This misstep undermines the credibility of the conclusions drawn by Glantz et al., especially when those conclusions are used to assert that cross-sectional and longitudinal studies produce comparable estimates of risk.

What makes these statistical errors particularly troubling is not merely that they occurred, but that they were embedded within a high-profile meta-analysis that has had a tangible impact on public understanding and policy development. The misuse of statistical tools in this context does not reflect a harmless misunderstanding. Rather, it represents a concern in scientific reasoning that can mislead readers, distort public understanding of risk, and skew the policy debate around e-cigarettes.

## Why this matters

The consequences of flawed meta-analyses are not confined to academic debate. Their impact ripples outward into public discourse, media narratives, clinical decision-making, and policy development. When statistical errors or methodological shortcuts shape the conclusions of high-profile reviews, these mistakes can have very real consequences for individuals trying to make informed decisions about their health. In the case of tobacco harm reduction, misleading or overstated evidence may deter smokers from switching to less harmful alternatives such as e-cigarettes. Subjective evidence indicates that e-cigarette users consistently rate their health more favorably than smokers [24, 25]. While self-reported health is not a substitute for clinical outcomes, these findings align with accumulating evidence that e-cigarettes are substantially less harmful than combustible tobacco products. This position has been affirmed by leading public health institutions, including the US National Academies of Science and Medicine, the UK Royal College of Physicians, and Public Health England, all of which have consistently emphasized the reduced relative risk of vaping compared to smoking [26–28].

When inaccurate or poorly constructed meta-analyses claim equivalency between the risks of smoking and vaping, they may unintentionally reinforce smoking behavior. Smokers who are considering switching may be deterred by alarmist headlines or seemingly authoritative conclusions that fail to distinguish between absolute and relative risks. This miscommunication is particularly damaging for populations that stand to benefit most from harm reduction strategies, including individuals who have struggled to quit smoking through other means.

Beyond the individual level, there are broader implications for public policy and scientific literacy. Flawed meta-analyses often become “anchor citations” that are repeatedly referenced in policy briefs, public health campaigns, media coverage, and even clinical guidelines. Once established, these citations can take on a life of their own, being treated as definitive summaries of the evidence even when their underlying data and interpretations are deeply problematic. As they are recycled across platforms and discussions, the opportunity to correct or contextualize their findings becomes increasingly difficult. This process can contribute to the persistence of misinformation within the evidence base, complicating efforts to establish a clear and accurate understanding of tobacco harm reduction. The scientific community must therefore take seriously the responsibility to ensure that meta-analyses, especially those with significant policy relevance, are methodologically robust, transparently reported, and appropriately interpreted.

## A call for meta-analytic reform

The case reviewed by Rodu and colleagues highlights more than a single flawed analysis; it reflects broader systemic challenges in how meta-analyses are conducted, reviewed, and interpreted, particularly in areas of scientific controversy such as e-cigarette research. As these analyses often carry considerable influence in shaping public health narratives, clinical recommendations, and regulatory actions, there is an urgent need for reform. Statistical sophistication cannot substitute for conceptual rigor, and superficial metrics must not be allowed to overshadow questions of clinical relevance, causal inference, and transparency. Editors, peer reviewers, and researchers share a collective responsibility to uphold higher standards. The credibility of science is at stake. To help achieve this, we propose a set of practical reforms aimed at improving the quality, credibility, and utility of future meta-analyses (See Table 1).

**Table 1:** Our recommendations

Reform	Description	Practical Implementation
Pre-registration of Protocols	Define disease categories, exposure definitions, and inclusion or exclusion criteria in advance to promote transparency and reduce selective reporting	Researchers should register meta-analysis protocols on platforms such as the Open Science Framework (OSF) or PROSPERO before data extraction begins. This ensures that analytical decisions are driven by scientific rationale rather than by data-driven convenience
Stricter Outcome Pooling	Only pool outcomes that share clear clinical and pathophysiological similarities. Aggregating unrelated conditions may generate unreliable or overstated conclusions and diminish the clinical utility of the findings	Authors must provide a justification for each pooled category and explicitly exclude outcomes that do not share a plausible mechanistic link. For example, erectile dysfunction should not be grouped with myocardial infarction under a single “cardiovascular disease” category without a compelling reason
Transparency in Data Handling	Many meta-analyses draw upon large-scale population datasets that are reused across multiple studies. Without clear disclosure, this can lead to inadvertent duplication, double-counting, and inflated weightings in pooled estimates	Researchers should provide full disclosure of all data sources and identify any overlaps across included studies. Journals should require that reused datasets such as PATH or NHIS be flagged, with sensitivity analyses conducted to assess the impact of potential duplication
Exposure Timing Consideration	Observational studies used in meta-analyses must account for the timing and duration of exposure relative to outcome onset. Without temporal sequencing, causal inferences are fundamentally flawed	Study designs should include detailed information about when participants began smoking or vaping, and when health outcomes were diagnosed. Meta-analysts should be required to assess whether included studies have sufficient temporal resolution to support causal interpretations

## Conclusion

The critique by Rodu and colleagues serves as a timely and important reminder that the strength of scientific conclusions depends not on the volume of data but on the integrity of the methods used to analyze and interpret it. Meta-analyses, when carefully constructed, have the potential to distill clarity from complexity. However, in the absence of conceptual rigor, transparent reporting, and methodological precision, they can just as easily amplify error, mislead public discourse, and misinform policy. In recent years, meta-analyses have come to occupy a privileged position in the evidence hierarchy, often regarded as the final word in scientific debates. Yet this status must not be taken for granted. Aggregating flawed or incomparable studies does not create strength through numbers. Instead, it creates the illusion of authority while concealing the structural weaknesses of the underlying evidence. The lesson from Rodu et al.’s analysis is not that meta-analysis should be abandoned, but that it must be reformed. Greater scrutiny must be placed on how studies are selected, how outcomes are classified, how exposure is measured, and how statistics are interpreted. These concerns have also been echoed in a new reanalysis by Lee and Farsalinos [29], who independently demonstrated major flaws in the Glantz et al. meta-analysis, reaffirming the urgent need for greater methodological integrity in this field.

## Data Availability

Not applicable.

## Declarations

### Patient and public involvement

Patients and/or the public were not involved in the design, or conduct, or reporting, or dissemination plans of this research.
